# GATK PathSeq: a customizable computational tool for the discovery and identification of microbial sequences in libraries from eukaryotic hosts

**DOI:** 10.1093/bioinformatics/bty501

**Published:** 2018-07-04

**Authors:** Mark A Walker, Chandra Sekhar Pedamallu, Akinyemi I Ojesina, Susan Bullman, Ted Sharpe, Christopher W Whelan, Matthew Meyerson

**Affiliations:** 1 Broad Institute of Massachusetts Institute of Technology and Harvard, Cambridge, MA, USA; 2 Department of Medical Oncology and Center for Cancer Genome Discovery, Dana-Farber Cancer Institute, Boston, MA, USA; 3 Department of Pathology, Harvard Medical School, Boston, MA, USA; 4 University of Alabama at Birmingham (UAB), Birmingham, AL, USA; 5 HudsonAlpha Institute for Biotechnology, Huntsville, AL, USA

## Abstract

**Summary:**

We present an updated version of our computational pipeline, PathSeq, for the discovery and identification of microbial sequences in genomic and transcriptomic libraries from eukaryotic hosts. This pipeline is available in the Genome Analysis Toolkit (GATK) as a suite of configurable tools that can report the microbial composition of DNA or RNA short-read sequencing samples and identify unknown sequences for downstream assembly of novel organisms. GATK PathSeq enables sample analysis in minutes at low cost. In addition, these tools are built with the GATK engine and Apache Spark framework, providing robust, rapid parallelization of read quality filtering, host subtraction and microbial alignment in workstation, cluster and cloud environments.

**Availability and implementation:**

These tools are available as a part of the GATK at https://github.com/broadinstitute/gatk.

**Supplementary information:**

Supplementary data are available at *Bioinformatics* online.

## 1 Introduction

Modern microbial genomic approaches utilize a combination of genomic technologies and bioinformatics tools to directly retrieve the genetic content of entire communities of organisms. Methods based on aligning sequencing reads to large databases of micro-organism genome references continue to illuminate the true microbial composition of deep sequenced biological samples. However, a limitation of such reference alignment-based analyses is that candidate pathogens may have limited identity to micro-organisms with sequenced genomes. To overcome this limitation, we pioneered the identification of novel non-human sequences (putative pathogens) in a comprehensive and unbiased manner using a ‘computational subtraction’ approach (Bhatt *et al.*, 2013; Kostic *et al.*, 2011; Weber *et al.*, 2002). This approach relies on the removal of host reads and analysis of the non-host portion of reads, resulting in the detection of both known and novel pathogens as well as any resident micro-organisms. Other methods for identifying novel micro-organisms in sequencing data from host organisms include digital transcript subtraction (Feng *et al.*, 2007), Pathoscope (Francis *et al.*, 2013), SURPI (Naccache *et al.*, 2014) and VirusSeq (Chen *et al.*, 2013).

We present an updated implementation of the PathSeq pipeline (Kostic *et al.*, 2011) that makes substantial improvements on the original version. First, computational efficiency has been improved by incorporating faster computational approaches (Materials and Methods Section). Second, unlike the original version, Genome Analysis Toolkit (GATK) PathSeq permits users to configure the workflow for multiple use cases such as different library types (i.e. whole-genome and RNA sequencing), sample types (e.g. blood, tissue, sputum, etc.), or host species. Third, the tool suite is implemented in Java with the GATK engine (McKenna *et al.*, 2010) and Apache Spark framework (Zaharia *et al.*, 2010), enabling parallelized data processing on local workstations, computing clusters and Google Cloud computing services (https://cloud.google.com).

## 2 Materials and methods

PathSeq begins with removal of low quality, low complexity, host-derived and duplicate reads (Supplementary Fig. S1, Supplementary Material S1). Several methodological improvements have been made to these steps in order to improve performance. Apache Spark is used to process batches of sequencing reads asynchronously in memory, thus maximizing resource utilization and minimizing slow hard-disk operations between pipeline stages. To accelerate filtering of low-complexity sequences, the RepeatMasker step (http://www.repeatmasker.org/) has been replaced with the symmetric DUST algorithm (Morgulis *et al.*, 2006). To rescue reads that are partially informative, the new version also incorporates trimming of low-quality bases, sequencing adapter artifacts and low-complexity sequences. The reads are then subjected to a fast *k*-mer search using a Bloom filter (Bloom, 1970) to detect short sequences from the host reference. Reads containing at least one host *k*-mer (*k* = 31) are removed. This step typically subtracts ∼90% of the host reads prior to performing sequence alignment, thus greatly reducing run time. The BLAST (Altschul *et al.*, 1990) aligner has been replaced with the faster BWA-MEM aligner (Li, 2013), which is used to map the remaining reads to the host reference (Supplementary Table S1).

PathSeq then aligns the remaining non-host reads to a reference of microbial genomes (viruses, bacteria, fungi, etc.; Supplementary Table S2) and classifies each read taxonomically. Alignment efficiency is again improved using BWA-MEM. Classification specificity has been increased by running the aligner in paired-end mode and requiring that both reads in a pair map to the same organism during classification. Finally, a report is generated containing microbial abundance estimates at each taxonomic level (e.g. species, genus, family, etc.).

## 3 Results

We used two datasets to demonstrate the performance of GATK PathSeq: (i) a total RNA sequencing library from HeLa cervical cancer cell line (Kostic *et al.*, 2011) and (ii) mRNA and WGS sequencing libraries from a cervical cancer patient (SGCX-NOR-030; Ojesina *et al.*, 2014). The cloud computing environment used for this study are described in Supplementary Material S2. A cutoff of 70% sequence identity was used for microbial read classification; this cutoff can be set by the user.

The HeLa library (10.3 million high-quality 76 bp paired-end reads) was used as a positive control and compared to the earlier PathSeq version. GATK PathSeq identifies 38 350 reads, comprising 0.37% of the library, aligned to HPV-18 (Supplementary Table S3), compared with the original PathSeq pipeline, which identified 25 879 HPV-18 reads (Kostic *et al.*, 2011). While we did not identify the cause of this increase, it may be due to improvements in the completeness of the microbe reference, read quality filtering and host read subtraction methodology. GATK PathSeq also reveals 4078 unmapped reads, or approximately 0.04%. The total CPU time taken for the analysis is 0.8 h.

The mRNA and WGS libraries derived from patient SGCX-NOR-030 contained approximately 218 million (76 bp) and 1.58 billion (101 bp) paired-end reads, respectively (Supplementary Material S3). These libraries were available as BAM files aligned to a human reference. Mapped reads were filtered before running PathSeq, reducing each library size by an order of magnitude. After quality filtering and host subtraction, the SGCX-NOR-030 mRNA and WGS samples contain 23 803 non-host reads and 2.2 million non-host reads including 1.7 million bacterial reads, respectively. GATK PathSeq identifies HPV-16 as the predominant HPV strain in SGCX-NOR-030 mRNA and WGS samples with read counts of 1984 and 9882 reads, respectively. This finding is consistent with previously published data (Ojesina *et al.*, 2014). The identity cutoff does not substantially affect the number of detected HPV reads, which only decreases by up to 13% if the identity cutoff is raised from 60% to 90% (Supplementary Table S4). Note that in both samples HPV-16 reads comprise <0.01% of the input reads and <0.001% of the original libraries, demonstrating PathSeq’s ability to detect extremely low abundance organisms. Residual unmapped reads were 12 215 (∼0.006% of the original reads) from the SGCX-NOR-030 mRNA library and 453 423 (∼0.03%) from the SGCX-NOR-030 WGS library (Supplementary Table S3). The total CPU time taken for analysis is approximately 2.3 h for the mRNA sample and 6.7 h for the WGS sample. Processing of large libraries scales efficiently in cluster environments (Supplementary Table S5).

Using a BLAST search, 36% of the unmapped reads of the HeLa library, 28% of the SGCX-NOR-030 mRNA library and 11% from the WGS library could be identified. These consisted primarily of human, HPV, bacteria, fungi and microbial eukaryote sequences (Supplementary Table S6) not represented in the PathSeq reference. Assembly of the unmapped reads using Fermi-lite (Li, 2015) yielded little further insight into the identity of these sequences in the HeLa and SGCX-NOR-030 mRNA libraries. However, in the SGCX-NOR-030 WGS library, 11 of the 20 largest assembled contigs mapped to *Sphingomonadaceae*, indicating the possible presence of a novel bacterium from this family (Supplementary Table S7). Understanding these residual unmapped reads will help us to improve GATK PathSeq and will also provide insight into the completeness of the human reference genome and the sequencing quality control process.

In summary, we have developed an adaptable and easily configurable pipeline for identification of microbial sequences in next generation sequencing data. This tool allows for customized analyses of biological samples with substantially reduced computational time.

## Supplementary Material

Supplementary Information
