# Supplementary material for: GATK PathSeq: a customizable computational tool for the discovery and identification of microbial sequences in libraries from eukaryotic hosts
Source: Bioinformatics. 2018 Jul 4;34(24):4287–9. doi: 10.1093/bioinformatics/bty501 (PMC6289130; doi:10.1093/bioinformatics/bty501)
Supplement: Supplementary Information [file bty501_supplementary_information.docx]

**Supplementary Materials**

GATK PathSeq: A customizable computational tool for the discovery and identification of microbial sequences in libraries from eukaryotic hosts.

Mark A Walker*, Chandra Sekhar Pedamallu*, Akinyemi I Ojesina, Susan Bullman, Ted Sharpe, Christopher W Whelan, and Matthew Meyerson

*Contributed equally to this work.

**Supplementary Methods**

**S1 Pipeline methods**

***S1.1 Filter low-quality and low-complexity reads***

Reads are first run through a series of filters to eliminate reads containing low-quality base calls or low-complexity sequences:

1. If the input is aligned and the --is-host-aligned flag is set, filter reads above a threshold identity score (matching bases less deletions) (score threshold 31)
2. Trim adapter sequences (minimum length of 12, maximum mismatches 1)
3. Remove reads with excessive A/T or G/C content (at least 29 A/T or C/G bases in a window of length 30, or at least 87 A/T or 89 C/G bases in a window of length 100)
4. Mask low-complexity sequence bases and base qualities using the sDUST algorithm (Liebert et al. 2006) (window size 64, threshold score 20)
5. Trim reads using base qualities
6. Remove reads below minimum length (2 bases shorter than original read length)
7. Mask low-quality bases (base quality PHRED threshold 15)
8. Remove reads with too many masked bases (ambiguous base threshold 2)
9. Remove sequence duplicates

***S1.2 Filter host reads***

Reads originating from the host are coarsely filtered by looking for short sequence matches using a fast k-mer search, which checks each consecutive k-mer in the read against the host k-mer library. In this study, the read is removed if there is at least one k-mer match (k = 31).

The reads are then aligned to the host-reference using the BWA-MEM algorithm. Reads with a sufficient identity score, defined as the number of matches less deletions, are removed.

The k-mer search is customizable. The k-mer library is built using the PathSeqBuildKmers tool, which finds the set of all unique k-mers in the host reference. The k-mers can be up to 31 bases in length and can be masked to allow mismatches at specific positions. In this study, the 16^th^ base was masked. The k-mer set can be represented using either a hash set or Bloom filter data structure. Bloom filters use less memory than hash sets but can also randomly give false positives and therefore may result in the erroneous removal of microbial reads. Users can adjust the false positive rate depending on tolerance for these errors. In tests, specifying a false positive rate of 0.001 resulted in <1% loss of microbial reads and reduced the k-mer library memory size ~3-fold. In this study, a Bloom filter of k-mers from the reference in Supplementary Table S1 with false positive probability of ~0.0001% was used.

***S1.3. Align to microbial reference***

The remaining reads are aligned to a microbial reference using the BWA-MEM algorithm. Paired-end alignment is used for pairs remaining after filtering.

***S1.4. Generate taxonomy report***

Alignments with sufficient identity score (in this study 70% of read length) are then used to estimate read counts and the relative abundance of microorganisms present in the sample at each level of the taxonomic tree (e.g. strain, species, genus, family, etc.). If a read maps to more than one organism, only the best alignment and any others with identity score within a margin of the best (in this study 2%) are retained. Scores are totaled for each taxon by summing the scores across all reads and the scores of any descendent taxa. Thus the score *S_t_* for taxon *t* is given by:

$$S_{t}=\left\{\begin{matrix} \frac{1}{L_{t}}\sum_{r\in R} \frac{\delta_{rt}}{n_{r}}+\sum_{d\in D_{t}} S_{d}\text{ if }L_{t}>0 \\ \sum_{d\in D_{t}} S_{d}\text{ otherwise} \end{matrix}\right.$$

where *L_t_* is the reference genome length of *t* in Mbp, *R* is the set of all reads, δ_rt_ is 1 if read *r* mapped to *t* and 0 otherwise, *n_r_* is the number of taxa that *r* mapped to, and *D_t_* is the set of descendants of *t*. Note that *L_t_* = 0 for higher taxa with no reference genome.

A normalized score is also reported, which by default is given as a percentage out of the taxon’s kingdom (e.g. bacteria, fungi, viruses, etc.). The total number of mapped reads and number of unambiguously mapped reads are also tabulated.

**S2 Benchmarking environment**

Unless otherwise noted, all samples were processed on a Google Cloud Compute virtual machine with 32 CPU cores, 208GB of memory, and 500GB SSD storage, costing $1.76 per hour. For the results shown in Supplementary Table S5, a Google Cloud DataProc cluster was configured with 16 virtual cores and 104 GB memory and 8 worker nodes each with 32 virtual cores, 208 GB memory, and 500 GB SSD storage.

**Supplementary Figures**


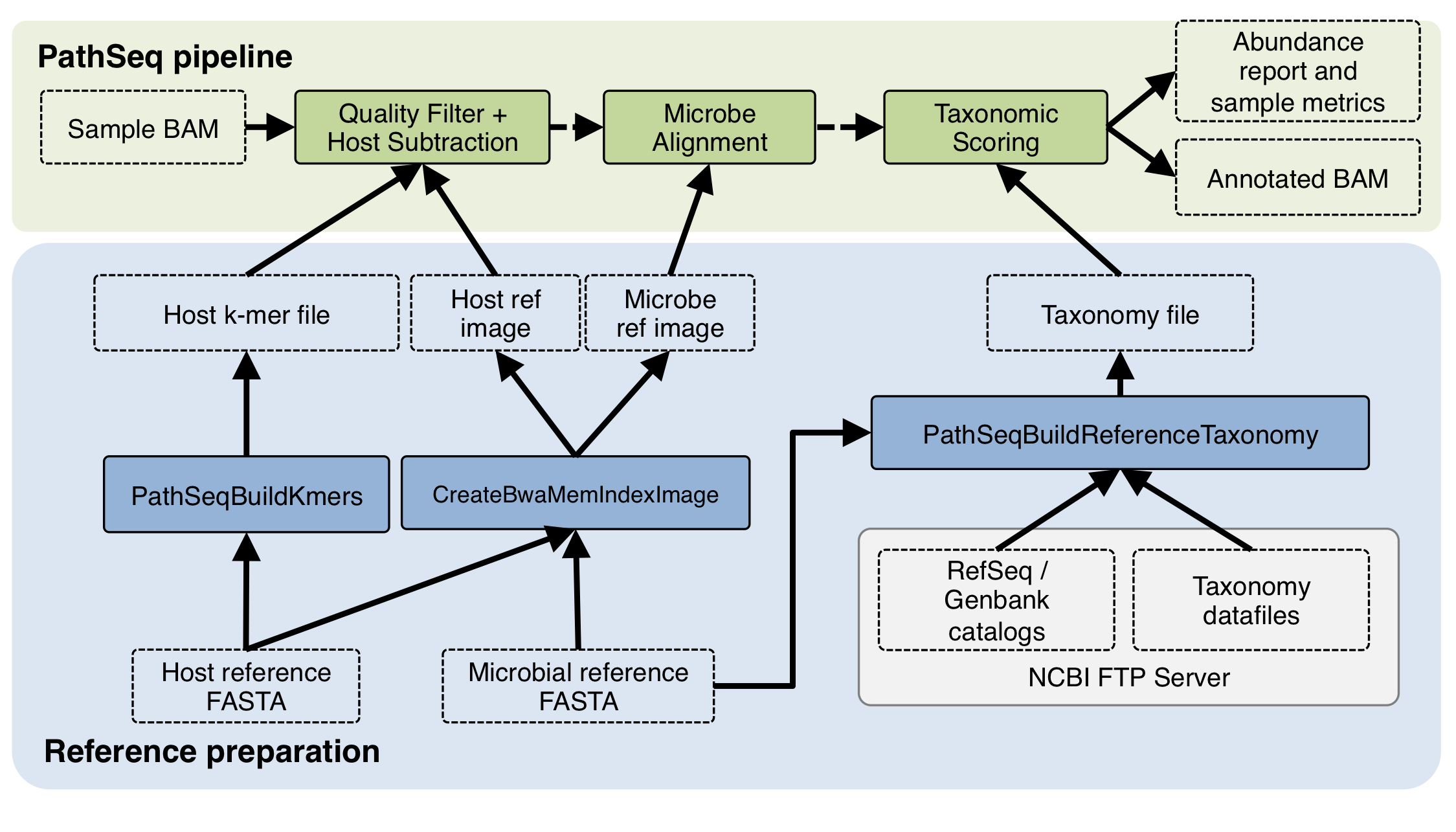


**Supplementary Figure S1. Schematic diagram of the GATK PathSeq pipeline and reference pre-processing steps.**. Note that the three PathSeq pipeline tools (filter, microbial alignment and microbial abundance) may be run with a single command using the PathSeqPipelineSpark tool. The k-mer, BWA index image creator, and taxonomy parser tools are also included in the GATK.

**Supplementary Tables**

| **Database** | **URL** | **Description** |
| --- | --- | --- |
| **hg38 human reference** | <https://genome.ucsc.edu/cgi-bin/hgGateway?db=hg38> | The latest human genome reference at the time of publishing. Includes main assembly, alternate contigs, decoys (not including EBV), and HLA sequences. |
| **Immuno Polymorphism Database (IPD) IMGT/HLA** | <http://www.ebi.ac.uk/ipd/imgt/hla/> | Highly variable sequences of the human major histocompatibility complex (MHC). |
| **NCBI UniVec** | <https://www.ncbi.nlm.nih.gov/tools/vecscreen/univec/> | Cloning vector sequences. |
| **Gencode (human v25)** | <https://www.gencodegenes.org/> | Curated database of human transcripts. |
| **GenBank accessions KY503218-KY5808060** | <https://www.ncbi.nlm.nih.gov/popset?DbFrom=nuccore&Cmd=Link&LinkName=nuccore_popset&IdsFromResult=1252311517> | Human breakpoint junction sequences. |

**Supplementary Table S1. Recommended list of human reference databases used in GATK PathSeq for human samples.**

| **Description** | **URL** |
| --- | --- |
| RefSeq archaea genomic sequences | <ftp://ftp.ncbi.nlm.nih.gov/refseq/release/archaea/> |
| RefSeq bacteria genomic sequences | <ftp://ftp.ncbi.nlm.nih.gov/genomes/refseq/bacteria/> |
| RefSeq fungi genomic sequences | <ftp://ftp.ncbi.nlm.nih.gov/refseq/release/fungi/> |
| RefSeq protozoa genomic sequences | <ftp://ftp.ncbi.nlm.nih.gov/refseq/release/protozoa/> |
| RefSeq viral genomic sequences | <ftp://ftp.ncbi.nlm.nih.gov/refseq/release/viral/> |

**Supplementary Table S2. Microbe reference database sources. Genome assemblies were combined to form a comprehensive microbial reference. For bacteria, one genome per species was used. This yielded a total microbial reference length of 65 Gbp. To filter spurious matches to low-quality unplaced contigs in draft genomes, alignments to non-virus contigs less than 2 kbp were filtered out.**

|  | **HeLa library** | **SGCX-NOR-030 mRNA** | **SGCX-NOR-030 WGS** |
| --- | --- | --- | --- |
| **Original sample reads** | 10,304,513 | 218,370,912 | 1,583,258,523 |
| **PathSeq input reads** | 10,304,513 | 26,527,539* | 113,482,301* |
| **Low-quality or low-complexity reads** | 578,896 | 20,050,697 | 90,103,588 |
| **Host reads** | 9,562,272 | 6,441,796 | 21,002,509 |
| **Duplicate reads** | 120,885 | 11,243 | 213,354 |
| **Micro-organism mapped reads** | 38,382 | 11,588 | 1,709,427 |
| **HPV mapped reads** | 38,350 | 1,984 | 9,882 |
| **Unmapped reads** | 4,078 | 12,215 | 453,423 |
| **Wall time (min)** | 1.57 | 4.45 | 12.62 |
| **CPU time (hr)** | 0.84 | 2.37 | 6.73 |
| **Estimated cost (USD)** | $0.05 | $0.13 | $0.37 |

*Number of reads that did not map to the hg19 human reference

**Supplementary Table S3. Summary of GATK PathSeq results on the HeLa library and patient SGCX-NOR-030 mRNA and WGS samples. Read counts at each stage of the pipeline and performance benchmarks are given. Note that the SGCX-NOR-030 libraries were previously aligned to the hg19 reference and were pre-filtered prior to running GATK PathSeq, which realigned the reads to hg38 for host read subtraction.**

| **Identity cutoff** | **HeLa library** | **SGCX-NOR-030 mRNA** | **SGCX-NOR-030 WGS** |
| --- | --- | --- | --- |
| 60% | 39,113 (0%) | 2,060 (0%) | 9,933 (0%) |
| 70% | 38,350 (-1.9%) | 1,984 (-3.7%) | 9,882 (-0.51%) |
| 80% | 37,719 (-3.6%) | 1,895 (-8.0%) | 9,797 (-1.4%) |
| 90% | 36,680 (-6.2%) | 1,799 (-13%) | 9,711 (-2.2%) |

**Supplementary Table S4. Number of reads identified as HPV at varying alignment identity cutoff values. Values in parentheses indicate reduction in detected reads as a percentage of those using the 60% identity cutoff.**

| **Spark Executors** | **HeLa library** | **SGCX-NOR-030 mRNA** | **SGCX-NOR-030 WGS** |
| --- | --- | --- | --- |
| 1 | 2.9 | 3.0 | 40 |
| 2 | 2.4 | 2.5 | 22 |
| 4 | 2.4 | 2.5 | 13 |

**Supplementary Table S5. Summary of GATK PathSeq approximate wall times (in minutes) running on a Google Cloud DataProc cluster. Each Spark executor utilized the resources of approximately one worker node. Poor scaling was observed for the smaller RNA libraries because the cost of local computations on each executor was small compared to overhead costs such as localizing the sequencing data, loading reference files into memory, and inter-node communication. The WGS library was more computationally demanding to process because it contained over 4x more total reads and two orders of magnitude more microbial reads, thus scaling well with additional executors.**

|  | **Name** | **Reads** |
| --- | --- | --- |
| **HeLa** | Human papillomavirus type 18 proteins E6 and E7 | 1,330 |
|  | Cyprinus carpio genome assembly | 149 |
|  | Homo sapiens cDNA clone IMAGE:5109562 (HPV18 chimera) | 74 |
|  | Homo sapiens isolate HGDP01412 mitochondrion | 30 |
|  | Human papillomavirus type 18 variant | 21 |
|  | Dioon edule genes for 5.8S rRNA, ITS2, 28S rRNA | 20 |
|  | Triticum aestivum chromosome 3B | 18 |
|  | Human papillomavirus type 18 isolate 1020994 E6 protein | 14 |
|  | PREDICTED: Clupea harengus uncharacterized mRNA | 13 |
|  | Diphyllobothrium latum genome assembly D_latum_Geneva | 13 |
| **SGCX-NOR-030 mRNA** | Human papillomavirus type 16 DNA | 548 |
|  | Uncultured Parvimonas sp. partial 16S rRNA gene | 523 |
|  | Cyprinus carpio genome assembly | 438 |
|  | Uncultured Rhodotorula genomic DNA | 422 |
|  | Rhodotorula mucilaginosa culture-collection | 374 |
|  | Rhodosporidiobolus colostri culture-collection | 279 |
|  | Uncultured bacterium partial 16S rRNA gene | 233 |
|  | Gymnopus perforans subsp. transatlanticus isolate | 188 |
|  | Uncultured eukaryote partial 18S rRNA gene | 172 |
|  | Homo sapiens MHC class I antigen (HLA-B) gene | 164 |
| **SGCX-NOR-030 WGS** | Spirometra erinaceieuropaei | 1,666 |
|  | Mitsuaria sp. 7 | 1,538 |
|  | Roseateles depolymerans strain KCTC 42856 | 1,249 |
|  | Human DNA sequence from clone RP11-380E6 | 1,195 |
|  | Homo sapiens FOSMID clone ABC16-1619H3 | 1,195 |
|  | Rubrivivax gelatinosus IL144 DNA | 1,044 |
|  | Paucibacter sp. KCTC 42545 | 954 |
|  | Leptothrix cholodnii SP-6 | 767 |
|  | Sphingomonas panacis strain DCY99 | 763 |
|  | Methylibium petroleiphilum PM1 | 722 |

**Supplementary Table S6. Summary of unmapped read alignments using BLAST on the nucleotide collection database (seed length 11, e-value cutoff of 10^-5^, no low-complexity reference masking). Each read was classified by the hit with the lowest e-value. In the case of a tie, the read was assigned to the sequence with the greatest number of hits. The top ten results for each sample are shown.**

|  | **Length (bp)** | **Top BLAST Hit** | **Reads** |
| --- | --- | --- | --- |
| **HeLa** | 219 | Human ORFeome Gateway entry vector pENTR223 | 40 |
|  | 207 | Homo sapiens cDNA clone IMAGE:5109562 (HPV18 chimera) | 361 |
|  | 153 | Homo sapiens cDNA clone IMAGE:5109562 (HPV18 chimera) | 8 |
|  | 139 | Human papillomavirus type 18 isolate HPV18Hap36 | 11 |
|  | 109 | Homo sapiens clone NA12878_chr19_1162210_1162211 | 5 |
|  | 107 | Homo sapiens ankyrin repeat and SOCS box-containing | 7 |
|  | 107 | Human papillomavirus type 18 isolate 83A.18 | 5 |
|  | 103 | Human papillomavirus type 18 isolate HPV18Hap38 | 66 |
|  | 101 | Human ORFeome Gateway entry vector pENTR223 | 6 |
|  | 88 | Human papilloma virus type 18 DNA | 9 |
| **SGCX-NOR-030 mRNA** | 641 | Uncultured fungus clone WMU_D12-15 28S | 96 |
|  | 387 | Heteromita globosa strain ATCC 50780 28S | 66 |
|  | 348 | Uncultured eukaryote clone nco54a08c1 18S | 34 |
|  | 335 | Plasmodiophora brassicae isolate Pyeongchang 2 18S | 45 |
|  | 313 | Sporobolomyces ficis culture-collection CBS:9205 | 137 |
|  | 310 | Uncultured fungus clone 23_NA3_P31_D5 18S | 49 |
|  | 287 | Uncultured Basidiomycota clone RP1_5_1E_21 | 19 |
|  | 270 | Heteromita globosa strain ATCC 50780 28S | 21 |
|  | 240 | Plasmodiophora brassicae isolate Pyeongchang 2 18S | 24 |
|  | 240 | Uncultured fungus clone D05E02 28S | 14 |
|  | 239 | Rhodotorula glutinis strain ATCC 16726 18S | 21 |
| **SGCX-NOR-030 WGS** | 21,998 | Blastomonas sp. RAC04 | 6,749 |
|  | 14,441 | Sphingobium cloacae plasmid pSCLO_5 DNA | 4,477 |
|  | 13,419 | Caulobacter sp. K31 | 4,335 |
|  | 11,709 | Sphingobium sp. TKS chromosome 1 | 4,266 |
|  | 9,503 | No hits | 3,055 |
|  | 9,396 | Novosphingobium resinovorum strain SA1 plasmid | 2,613 |
|  | 8,962 | Novosphingobium resinovorum strain SA1 plasmid | 2,763 |
|  | 8,225 | Pseudomonas citronellolis strain SJTE-3 | 2,978 |
|  | 7,071 | Sphingopyxis fribergensis strain Kp5.2 | 2,402 |
|  | 5,287 | Sphingopyxis macrogoltabida strain 203N | 1,460 |
|  | 4,902 | Sphingobium sp. MI1205 chromosome 1 | 1,420 |
|  | 4,721 | Agrobacterium vitis S4 plasmid pAtS4c | 505 |
|  | 4,583 | Sphingobium chlorophenolicum L-1 chromosome 2 | 1,292 |
|  | 4,339 | No hits | 469 |
|  | 3,951 | Novosphingobium pentaromativorans US6-1 plasmid | 895 |
|  | 3,790 | Acidovorax sp. RAC01 | 297 |
|  | 3,717 | No hits | 394 |
|  | 3,629 | Sphingobium sp. TKS plasmid pTK5 | 1,099 |
|  | 3,013 | Methylobacterium extorquens AM1 megaplasmid | 422 |
|  | 2,995 | Sphingomonas panacis strain DCY99 | 909 |
|  | 2,788 | Methylibium petroleiphilum PM1 plasmid RPME01, | 179 |

**Supplementary Table S7. Summary of contigs assembled from unmapped reads. The top 10 largest contigs are shown for the HeLa and SGCX-NOR-030 mRNA samples, and the top 20 are shown for the SGCX-NOR-030 WGS sample. Contigs were aligned using the BLAST search described in Supplementary Table S6. The right-hand column gives the number of unmapped reads that aligned to each contig.**

**References**

Liebert, M. A. et al. (2006). A Fast and Symmetric DUST Implementation to Mask Low-Complexity DNA Sequences. J. Comput. Biol., 13**,** 1028–1040.
